# Factors promoting willingness to practice medicine in rural regions and awareness of rural regions in the university’s catchment area – cross-sectional survey among medical students in central Germany

**DOI:** 10.3205/zma001634

**Published:** 2023-06-15

**Authors:** Christine Brütting, Sabine Herget, Felix Bauch, Melanie Nafziger, Anja Klingenberg, Tobias Deutsch, Thomas Frese

**Affiliations:** 1 University of Halle-Wittenberg, Faculty of Medicine, Institute for General Medicine, Halle (Saale), Germany; 2 University of Leipzig, Faculty of Medicine, Department of General Practice, Leipzig, Germany; 3 aQua Institute for Applied Quality Improvement and Research in Health Care, Göttingen, Germany

**Keywords:** physician shortage, curriculum development, rural areas, student survey

## Abstract

**Aim:**

Many universities offer rural medical internships for medical students. The present survey was designed to show how rural medical work is perceived by students, whether these perceptions are associated with origin and previous experience, and how well medical students know rural regions in the vicinity of their university. In addition, students were asked how to support and inspire medical students to later work in a rural region.

**Methods:**

This cross-sectional study was based on an anonymous online survey of medical students at the Universities of Halle-Wittenberg and Leipzig. The evaluations included descriptive statistics, statistical group comparisons, and qualitative content analysis of free text answers.

**Results:**

A total of 882 students took part in the survey. Students who had grown up in a rural region or had lived there for a longer time (71.7% of the respondents) rated the work-life balance better (p<0.01) and the patient variety in the countryside slightly higher (p<0.05) than their fellow students from the big city. Students who had worked in a rural practice or hospital before (62.2%) rated patient diversity (p<0.001) and work variety (p<0.001), as well as workload (p<0.01), slightly higher in rural areas than students with no prior experience. On average, the specified rural model regions were still unknown to more than 60% of the students. The suggestions for attracting medical students to later work as rural physicians included financial incentives and, above all, better information about life as a rural physician and the rural regions.

**Conclusion:**

Thus, the medical faculties of the universities as well as the counties threatened by medical undersupply should further expand the transfer of knowledge and experience regarding rural physician life for the students.

## Zusammenfassung

**Zielsetzung:**

Viele Universitäten bieten landärztliche Praktika für Studierende an. Die vorliegende Umfrage sollte zeigen, wie die ärztliche Tätigkeit auf dem Land von Studierenden wahrgenommen wird, ob diese Wahrnehmungen mit Herkunft und Vorerfahrungen assoziiert sind und wie gut Medizinstudierende ländliche Regionen im Umfeld ihrer Universität kennen. Zudem wurden die Studierenden gefragt, wie man Medizinstudierende dabei unterstützen und dafür begeistern kann, später in einer ländlichen Region zu arbeiten.

**Methodik:**

Die Querschnittstudie basiert auf einer anonymen Online-Umfrage der Medizinstudierenden der Universitäten Halle-Wittenberg und Leipzig. Die Auswertungen beinhalteten deskriptive Statistik, statistische Gruppenvergleiche sowie qualitativ-inhaltsanalytische Analysen von Freitextantworten.

**Ergebnisse:**

Insgesamt nahmen 882 Studierende an der Umfrage teil. Studierende, die in einer ländlichen Region aufgewachsen waren oder länger dort gelebt hatten (71,7% der Befragten), schätzten die Work-Life-Balance (p<0,01) besser und die Patient*innenvielfalt (p<0,05) auf dem Land etwas höher ein als ihre Kommiliton*innen aus der Großstadt. Studierende, die schon einmal in einer Praxis oder einem Krankenhaus in einer ländlichen Region gearbeitet hatten (62,2%), schätzten die Patient*innenvielfalt (p<0,001) und die Abwechslung in der Arbeit (p<0,001), aber auch die Arbeitsbelastung (p<0,01) auf dem Land etwas höher ein als Studierende ohne Vorerfahrung. Die Modellregionen waren im Durchschnitt mehr als 60 % der Studierenden noch unbekannt. Die Vorschläge zur Gewinnung von Medizinstudierenden für eine spätere landärztliche Tätigkeit umfassten neben finanziellen Anreizen vor allem eine bessere Informationsvermittlung über das Landärzt*inleben und die ländlichen Regionen.

**Schlussfolgerung:**

Universitäten und die von ärztlicher Unterversorgung bedrohten Landkreise sollten die Wissens- und vor allem die Erfahrungsvermittlung bezüglich des Landärzt*inlebens für die Studierenden weiter ausbauen.

## Introduction

In Germany, as well as in many other Western countries, an increasing shortage of physicians is becoming apparent, especially in rural regions [1], [2], [3]. Various factors can contribute to medical students deciding to work as a rural physician [4]. A personal connection to the rural region is particularly important. For example, medical students who grew up primarily in a rural region are more likely to feel a connection to that region [5]. Regional internships are one way to promote the connection to rural regions [6], [7].

**Figure 1:**
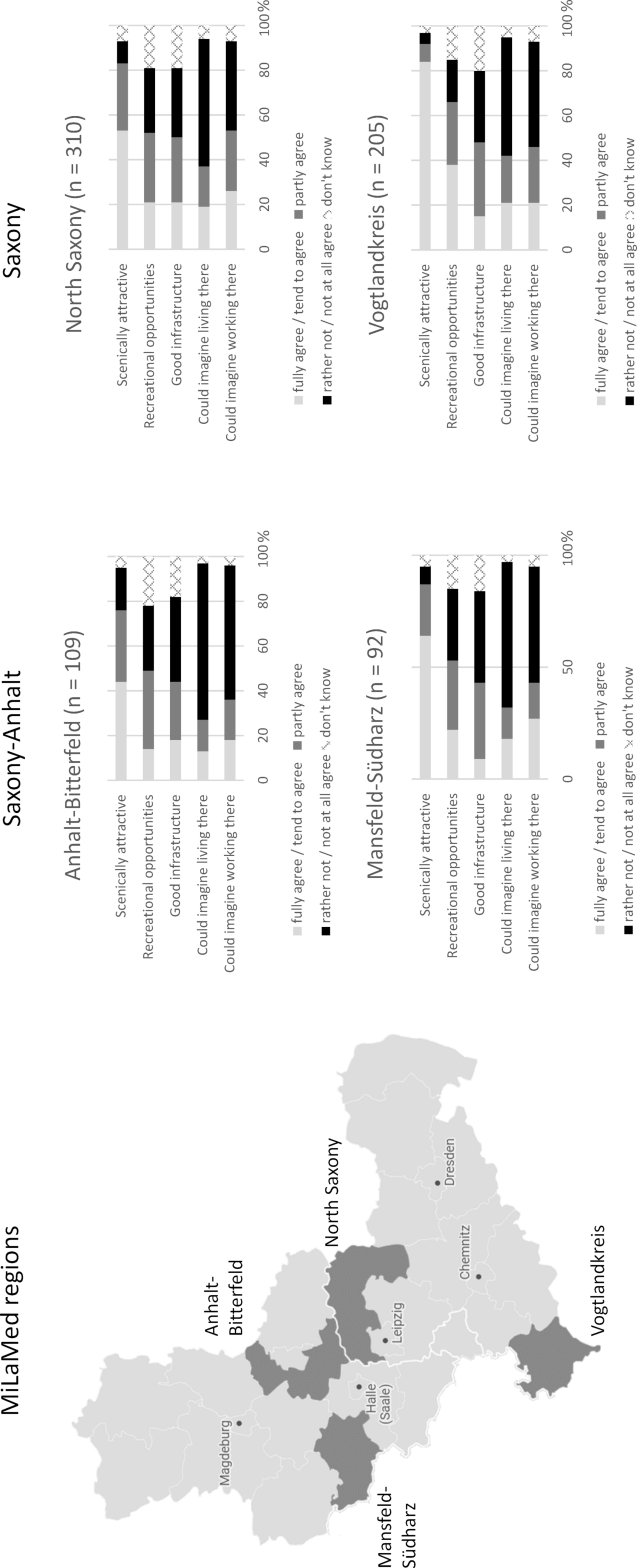
Overview of Central Germany with the model regions Anhalt-Bitterfeld and Mansfeld-Südharz in Saxony-Anhalt as well as North Saxony and Vogtlandkreis in Saxony, which are undersupplied with rural physicians, and assessments of the 4 model regions by students who had already been to the regions at least once or for a longer time or who had lived or worked there.

MiLaMed (“Central German Concept for the Longitudinal Integration of Rural Medical Training Content and Experience in Medical Studies”) is a cooperative teaching project of the Martin Luther University Halle-Wittenberg and the University of Leipzig with the aim of establishing a longitudinal curriculum for care in rural areas [https://www.milamed.de]. The concept includes teaching content implemented in the university compulsory and elective curriculum, supplementary online teaching content to accompany rural placements, and targeted support and promotion of placements in 4 model regions affected by undersupply (see figure 1 (Fig. 1)). In this context, the support offers cover all essential internships of medical studies in Germany, such as nursing internships, clinical traineeships, or final clinical year in different specialties. In addition, students are supported in their search for overnight accommodations and the costs for travel and lodging are covered. The participating districts provide a leisure allowance for longer internship stays.

The trial phase of MiLaMed began in April 2020 and was evaluated by aQua Institute for Applied Quality Improvement and Research in Health Care (Göttingen). At the beginning of the trial phase, a survey was conducted among all medical students at both participating university sites to describe their initial situations before the new services were made available. Students were asked how they assessed medical work in a rural region compared with a large city, to what extent they were familiar with 2 rural-small town districts in the catchment area of the university, and how they assessed them in terms of scenic attractiveness, available recreational opportunities, and infrastructure. In addition, the students were asked how, in their view, medical students could be supported and persuaded to later practice medicine in a rural-small town region.

## Methods

### Study design and population

The cross-sectional survey was conducted online and anonymously between April 8 and May 18, 2020. The link to the questionnaire as well as a cover letter explaining the study background were sent to all students of human medicine at the Universities of Leipzig and Halle-Wittenberg by e-mail from a central location. Approximately 1380 students (63.1% female) were enrolled in human medicine at the University of Halle and 2250 students (65.9% female) at the University of Leipzig in the summer semester 2020. A valid e-mail address was available to our institute from 1217 medical students in Halle and from 1868 in Leipzig. To increase the response rate, 2 reminder e-mails (after 3 and 5 weeks) were sent. In addition, attention was drawn to the survey in the respective student portals.

#### Instrument

The questionnaire was developed by the aQua Institute for Applied Quality Improvement and Research in Health Care (Göttingen) in cooperation with the departments of general medicine in Halle and Leipzig in an interdisciplinary team. In addition to sociodemographic data, previous professional and internship experiences, attitudes toward a later medical career in urban or small-town rural areas, comparative assessments of urban and rural medical activity, as well as knowledge and assessments of the MiLaMed model regions of the respective location were recorded. In free text format, the survey asked how medical students could be supported or recruited with regard to later medical practice in rural-small town areas. Before conducting the main survey, the questionnaire was optimized on the basis of a pre-test with 17 students from both universities (12 female, 5 male).

#### Data analysis

Statistical analyses were performed using IBM SPSS Statistics 25. To account for missing values in individual items, frequencies are presented as % (n/n_valid_). For metric variables, the arithmetic mean and standard deviation were given.

In the student survey, students could choose between “big city”, “small town”, and “country” for the question of origin. Because the attitudes of students from the small town and the country differed only marginally, we considered these 2 groups as one group in our analysis.

Assessments of physician employment in rural areas compared with large cities could be given on a 5-point scale (much lower, somewhat lower, about the same, somewhat higher, much higher). For the assessments in question, in addition to the descriptive analysis across the entire sample group, group comparisons were made with regard to the variables “origin” (rural/small town vs. large city) as well as “experience with rural medical practice” (experience vs. no experience). The chi-square test was used for frequency comparisons, and the Mann-Whitney U test was used for comparisons of central tendency (in the absence of normal distribution according to the Kolmogorov-Smirnov test). Significance was assumed for a probability of error of p<0.05.

The free text answers of the students were categorized according to the method of content-structuring and quantifying qualitative content analysis according to Mayring [8]. For this purpose, after reviewing all the material, 2 independent researchers inductively created category systems, which were then compared and adjusted in a consensus process. Afterwards, all answers were assigned to these categories. The additional assignment by a third, previously uninvolved scientist resulted in a random-corrected interrater reliability of 0.91 (Cohen’s K). Finally, absolute and relative frequencies were calculated for all categories.

## Results

### Sample description

Of the 3085 students contacted, 882 participated in the survey (response rate=28.6%). The average age of the respondents was 24.0 years, 70.7% (624/882) were female, and for almost two thirds a rural region was generally considered for a later medical career (see table 1 (Tab. 1)). Students who had grown up in a rural region or had lived there for a long time (632/882) were more likely to imagine working as a physician in a rural area later in life than students who grew up in a large city (72.6% vs. 37.6%, p<0.001). Students who had previously worked in a practice or hospital in a rural area (549/882) were more likely to imagine working as a physician in a rural area later in life than students who did not have such prior experience (74.7% vs. 42.9%, p<0.001).

#### Perception of medical activity in rural regions

**Figure 2:**
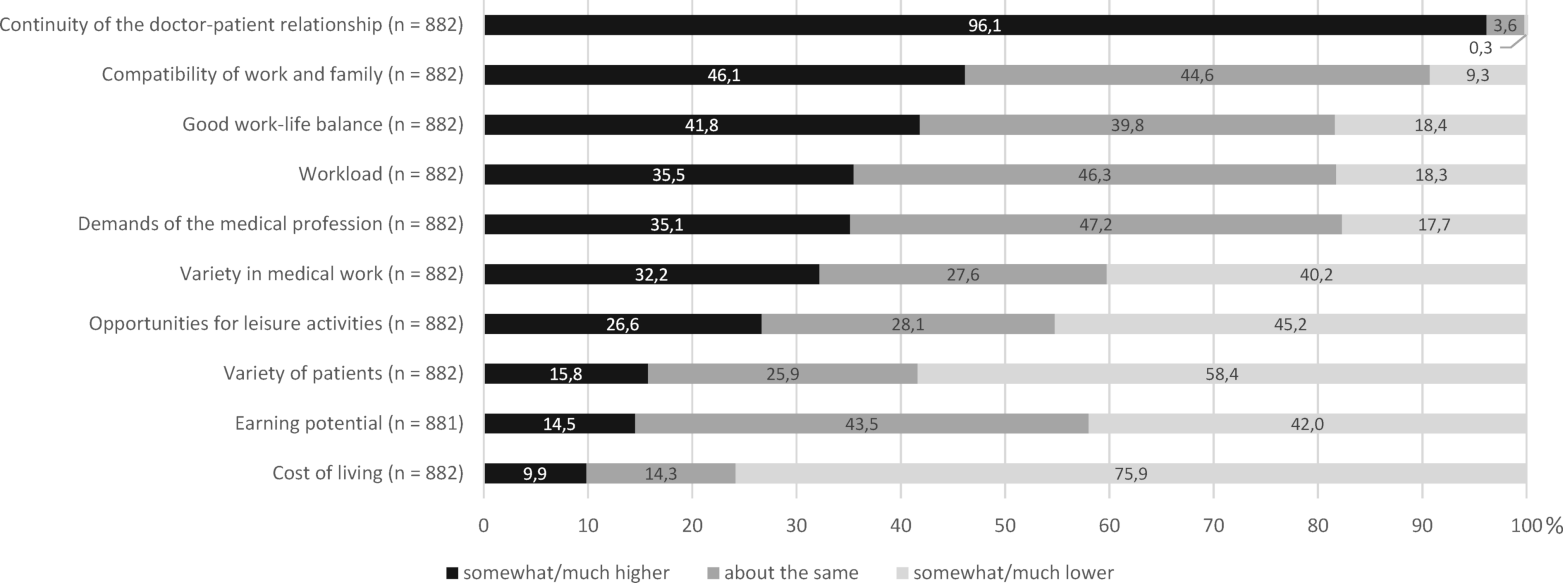
Assessment of medical activity and living situation in rural region compared with metropolitan location.

Almost all respondents assumed greater continuity in the doctor-patient relationship in rural regions compared with the big city. The compatibility of work and family, the demands of the medical profession, and the possibility of a good work-life balance were on average rated higher in a rural region than in a large city, and the cost of living rate was lower. On the other hand, the diversity of patients, the variety of medical activities, the earning potential, and the opportunities for leisure activities were rated somewhat lower on average in a rural region and the workload higher than in a metropolitan location (see figure 2 (Fig. 2)).

**Figure 3:**
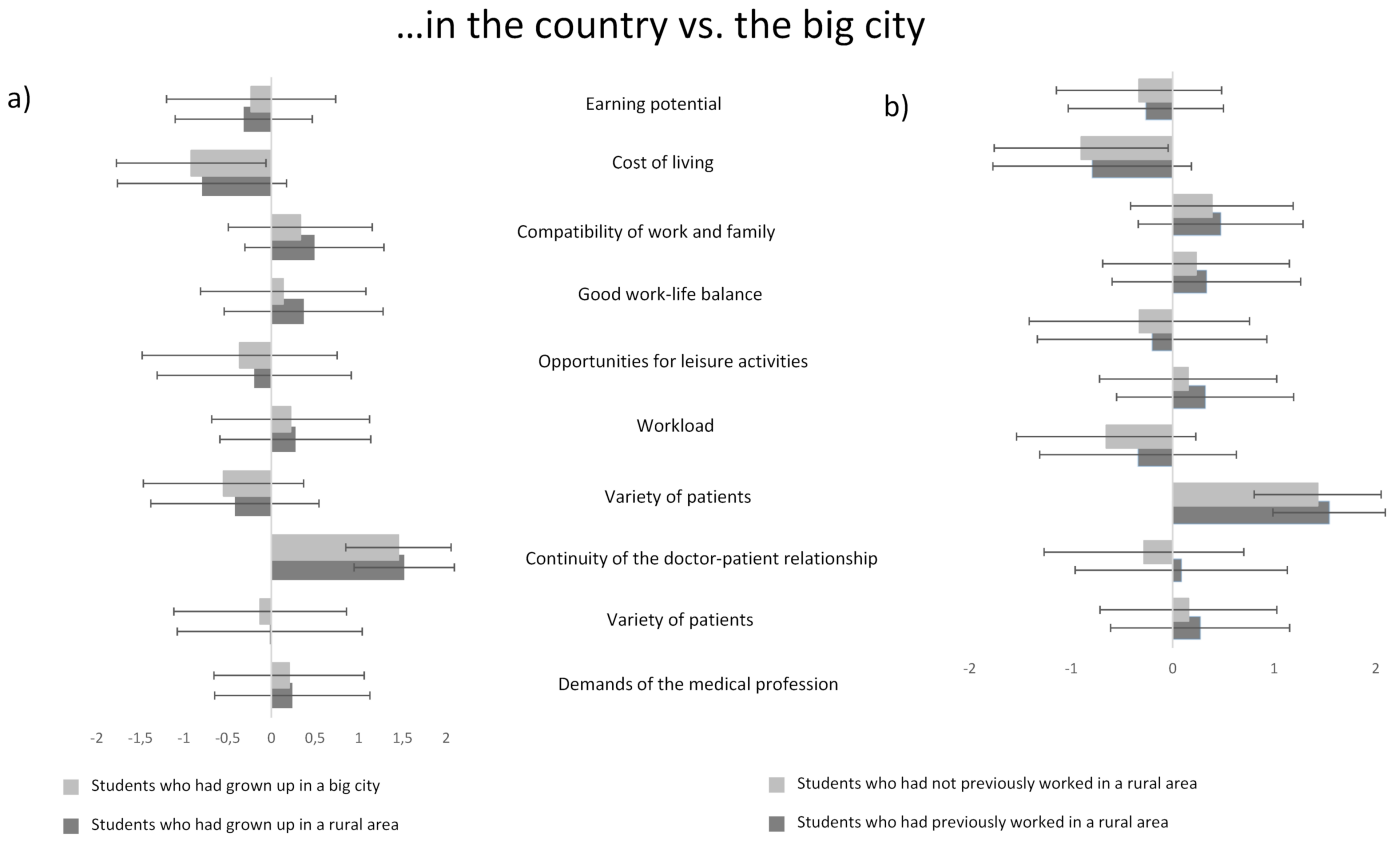
Mean values (on a scale of -2=much lower, -1=somewhat lower, 0=about the same, 1=somewhat higher, 2=much higher) and standard deviations of the assessed aspects of working as a physician in rural regions compared with a metropolitan location, assessed by the following: a) Students who grew up in a small town/rural area or lived there for a longer time (n=632) and students who grew up in a big city (n=280) b) Students with previous work experience in a rural area (n=549) and students without work experience in a rural area (n=333).

Students who had grown up in a rural region (632/882) rated the work-life balance (p<0.01) better and the compatibility of work and family (p<0.05) as well as the variety of patients in the countryside (p<0.05) slightly higher than their fellow students from the big city (see figure 3a (Fig. 3)). Students who had already worked in a practice or hospital in a rural region (549/882) rated the diversity of patients (p<0.001), the variety in medical work (p<0.001), and the continuity of the doctor-patient relationship (p<0.05), but also the workload (p<0.01) in a medical job in the countryside compared with the big city somewhat higher than students without such experience (see figure 3b (Fig. 3)).

Among students who had grown up in a rural region, 72.8% had ever worked in a rural region in a practice or hospital (460/632). Of the students who had grown up in a large city, as many as 35.6% (89/250) had worked in a rural region in a practice or hospital.

#### Awareness of rural regions

Of the students from Halle, 66.4% (182/274) did not know or had only heard or read about the Mansfeld-Südharz region and 60.2% (165/274) the Anhalt-Bitterfeld region. In comparison, 49.0% (298/608) of the students from Leipzig did not know the region of North Saxony and 66.3% (403/608) did not know the region of Vogtlandkreis or had only heard or read about it. 

Many students described the rural districts as scenically attractive. In contrast, the recreational opportunities according to their own interests and the infrastructure, were rated significantly lower on average. After all, about 20-25% of the students who had already been to one of the model regions at least once could imagine working as a physician in these regions. Almost 20% of the students could even imagine living there as well. Interestingly, in the categories of recreational opportunities and infrastructure, about 20% of the students could not give an assessment on this because they lacked the relevant knowledge (see figure 1 (Fig. 1)).

#### Suggestions for attracting medical students to later work as rural physicians

Participants were asked for their views on how to support and inspire medical students to later work in a rural-small town region. Here, 470 of 882 students had used the option of free text answers. Thus, a total of 1227 answers could be grouped into 9 categories and 22 subcategories (see table 2 (Tab. 2)). In addition to financial incentives (e.g., rural physician supplement or scholarship programs as well as funded internships in rural areas) and more information about rural physician life during studies (e.g., exchange of experiences with rural physicians, summer schools, information about establishing), there were also suggestions about the regions (e.g., more information about the medical and cultural diversity there, improved infrastructure, the possibility of employment instead of establishing).

## Discussion

### Perception of medical activity in rural regions

Compared with working as a doctor in the big city, many students see greater continuity in the doctor-patient relationship, a better work-life balance, higher demands of the medical work, but also a higher workload in the context of working in rural areas. On the other hand, variety in the work and patient diversity are rated as lower, as are earning potential and opportunities for leisure activities (see figure 2 (Fig. 2)). However, some of these aspects are prejudices. For example, it is known from the literature that most rural physicians perceive their work as very varied [9]. In addition, the income of rural physicians is on average even higher than that of their colleagues in the city [10]. Reportedly, rural doctors treat more patients on average [11], [12].

Although both a rural origin and previous experience with medical work in the countryside lead students to be more likely to imagine working as a rural doctor, the assessment of individual aspects of rural medical work (including prejudices) does not differ very much from that of students from the big city or without previous experience, interestingly enough (see figure 3 (Fig. 3)). It is possible that the experience of several internships is needed to reduce prejudices. For example, a long-term observation of Australian medical students showed that attitudes toward rural medicine changed progressively with the number of rural internships [13]. In addition, the positive influence of mentors became more apparent the further students progressed in their rural clinical rotations [13]. This is where projects such as MiLaMed could come in, e.g., to reduce prejudices through contact between students and rural physicians and by promoting internships in rural regions.

#### Awareness of rural regions

Only about one-third to one-half of the students knew the small-town rural model regions in the direct catchment area of their university. Regions that are close to the place of study seem to be better known. For example, more than half of the Leipzig students surveyed had already been to the district of North Saxony, which is directly adjacent to the city of Leipzig (see figure 1 (Fig. 1)) and can be visited free of charge by students with the semester ticket. If one takes a closer look at the (general) medical care in the district of North Saxony, the former districts of Delitzsch and Eilenburg, which are directly adjacent to Leipzig, also have a significantly lower shortage of doctors, in contrast to Torgau, which is further away [14]. This pattern of better access to medical care in more urban regions has also been shown for Germany as a whole [15].

For about a quarter of the students who had already been to one of the model regions at least once, there was a general willingness to practice medicine in these counties later on. Within the overall sample, about three-fifths of respondents could imagine working in a rural region. Thus, the significantly lower numbers for the specific model regions could well be related to the corresponding regions. Many students come from other federal states due to the central allocation procedure for study places in medicine and may be more likely to imagine working in the vicinity of their home region later on.

In the districts of North Saxony, Anhalt-Bitterfeld, and Mansfeld-Südharz, students are more likely to imagine working there than living there. In contrast, students can imagine the Voigtlandkreis as a potential center of life in the same way as a center of work. The reason for this is possibly the long commute time. It takes about 1.5 to 2 hours to get from Leipzig to the district, both by car and by train. However, it is known from previous surveys of medical students that virtually no one is willing to invest more than one hour of commuting time [16]. In addition, the Vogtlandkreis is perceived to be by far the most attractive in terms of scenery, and it also performs twice as well in terms of recreational opportunities compared with the other regions (see figure 1 (Fig. 1)).

Interestingly, however, about 20% of the students who have already been to the model regions have information gaps, especially in the areas of recreational opportunities and infrastructure. This is another area where projects like MiLaMed can help to provide students with targeted information.

#### Suggestions for attracting medical students to later work as rural physicians

The results of the qualitative analysis of the students' recommendations with regard to motivating them to later work as physicians in rural areas emphasize different aspects. In addition to financial incentives (such as higher remuneration for rural medical activities or financial support and free accommodation for internships), students highlight in particular the importance of information and content on rural medical topics during studies as well as making medical and cultural diversity in rural regions visible and tangible (see table 2 (Tab. 2)). With these suggestions, it is noticeable that MiLaMed with its conceptual approach can serve to provide very much of that already. The present student survey took place directly at the beginning of the trial phase of the MiLaMed project. Therefore, the comparison of the results of the initial survey presented here with the results of a second online survey, 2 years after the start of the project, is of particular interest. This comparison will show in what respects the MiLaMed concept was successful and where it could still be optimized, if necessary.

## Strengths and limitations

The present study deals with an important topic of high relevance for health policy. The participation of 882 students from all 6 years of study at 2 medical faculties supports the validity of the results. The overall response rate of 28.6% can be considered good in the context of voluntary online surveys, and the distribution of important sample characteristics such as age and gender appears representative of medical students in Germany at the time of the survey [17]. However, some limitations should be considered when interpreting the results. It is true that medical students were explicitly motivated in the cover letter for the online survey to participate in the survey regardless of their attitude toward possible future work as a rural physician. Nevertheless, it cannot be ruled out that students who are interested in and open to this topic may have been more likely to participate than others. Therefore, it is possible that among survey participants, attitudes toward becoming a physician in a rural region are more positive than the average for all medical students. In addition, we cannot say whether the preferences expressed by the students in the free texts actually lead to a medical practice in a rural area or whether, at the time of a possible settlement, other factors do not additionally speak for or against a settlement in a rural area. Long-term observations or before-and-after surveys would be necessary for this purpose.

## Funding

The MiLaMed project is funded by the German Federal Ministry of Health (funding code: ZMVI1-2520FEP002).

## Competing interests

The authors declare that they have no competing interests. 

## Figures and Tables

**Table 1:**
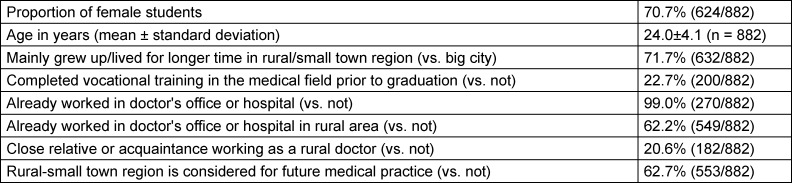
Sample description of students participating in the survey (n=882)

**Table 2:**
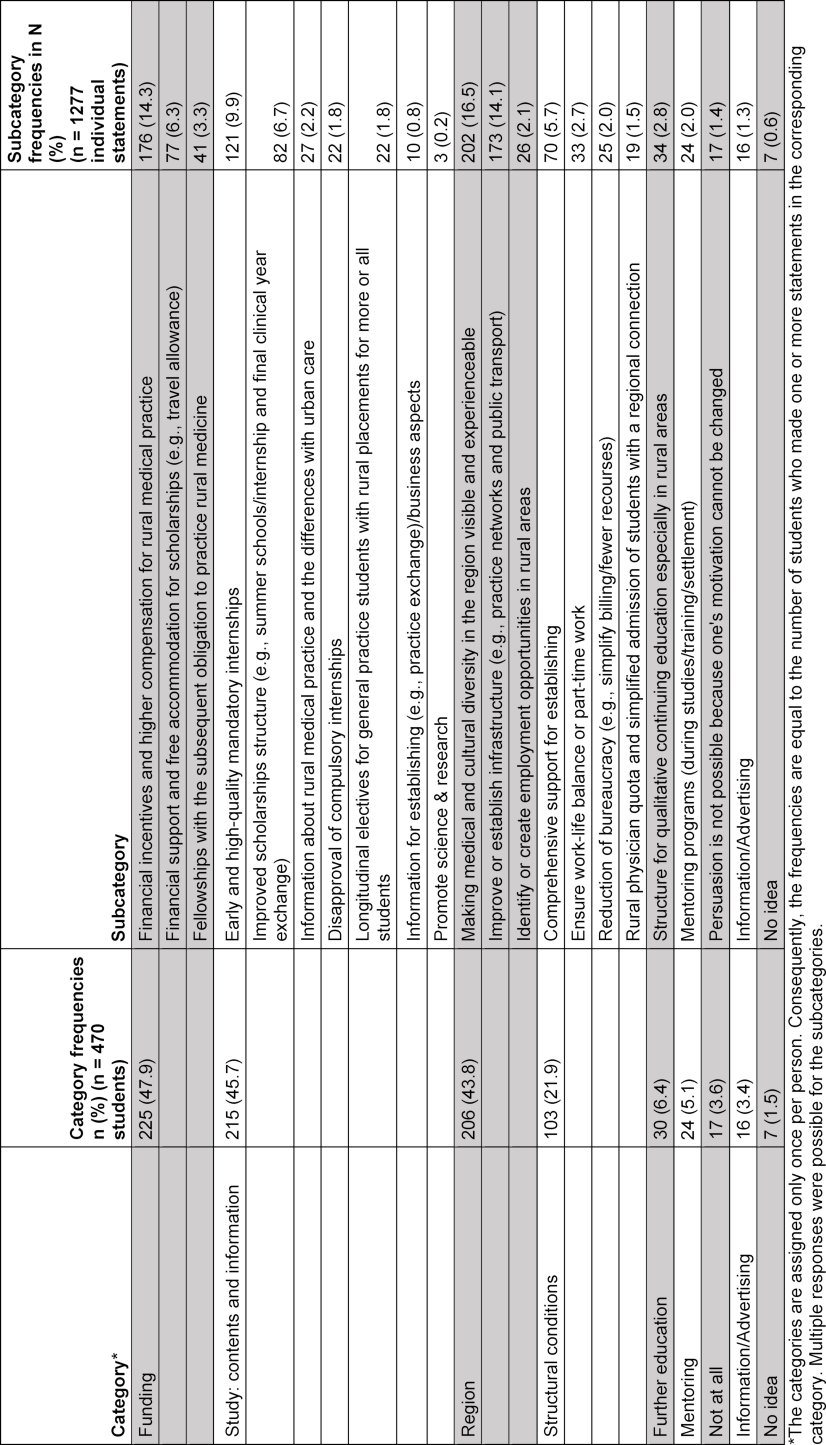
Analysis of n=1277 free text answers from n=470 students to the following question: *How do you think medical students could be supported and recruited to practice medicine in a rural-small town region?*
